# Supplementary material for: Integrating a Luminescent Porous Aromatic Framework into Indicator Papers for Facile, Rapid, and Selective Detection of Nitro Compounds
Source: Molecules. 2022 Sep 22;27(19):6252. doi: 10.3390/molecules27196252 (PMC9572729; doi:10.3390/molecules27196252)
Supplement: Supplementary file 1 [file molecules-27-06252-s001.zip › molecules-1919344-supplementary.pdf]

Supporting Information

Integrating a luminescent porous aromatic framework into indicator papers for facile, rapid, and selective detection of nitro compounds

Bo Cui [1], Changyuan Gao [2], Jiating Fan [1], Jinni Liu [1], Bin Feng [1], Xianghui Ruan [3], Yajie Yang [3], Ye Yuan [3], Kuo Chu [2*], Zhuojun Yan [1*], and Lixin Xia [1,4*]

1 College of Chemistry, Liaoning University, Shenyang 110036, China

2 School of Environmental Science, Liaoning University, Shenyang 110036, China

3 Key Laboratory of Polyoxometalate and Reticular Material Chemistry of Ministry of Education, Faculty of Chemistry, Northeast Normal University, Changchun 130024, China

4 Liaoning Key Laboratory of Chemical Additive Synthesis and Separation, Yingkou Institute of Technology, Yingkou 115014, China

Bo Cui and Changyuan Gao contributed equally to this work.

*Correspondence: chukuolnu@163.com (K.C.); zjyan@lnu.edu.cn (Z.J.Y.); lixinxia@lnu.edu.cn (L.X.X.)

1.1 Instruments

Fourier transform infrared (FT-IR) spectra were recorded in the range of 400-4000 cm^{-1} on an IR-Prestige 21 (Shimadzu Corporation, Japan) spectrometer with KBr pellets. The solid-state ^{13}C cross-polarization/magic-angle spinning (CP/MAS) NMR spectra were collected a Bruker AV ANCE III 400 MHz NMR. Powder X-ray diffraction (PXRD) patterns were conducted on a Bruker D8 ADVANCE diffractometer using Cu-Kα radiation (λ = 0.15418 nm) in a 2θ range of 4-40° at room temperature. Japanese Hitachi Su8010 scanning electron microscope (SEM) and Japan Electronics JEM-2100 transmission electron microscope (TEM) were adopted to investigate the morphology and micro-structure of PAF network. Thermogravimetric analyses (TGA) were performed on a Perkin-Elmer TGA-7 thermogravimetric analyzer from room temperature to 1000 ℃ in nitrogen atmosphere with a heating rate of 10 °C min^{-1}. N_2 adsorption-desorption isotherm at 77 K was measured using a Quantachrome AsiQ-C instrument. Ultraviolet spectrum in the range of 200-750 nm was recorded by the American Perkin-Elmer L35 ultraviolet spectrophotometer (UV). Fluorescence spectrum was obtained by the American VARIAN company FL750 fluorescence spectrometer (CARY Eclipse). The measurements of quantum yields were conducted on Fluoreomax-4-TCSPC with the integration sphere model. HOMO and LUMO calculations for PAFs and nitro compounds. All the molecular orbital calculations were performed with the Gaussian 09 D.01 program at the B3LYP/6-31G* level.

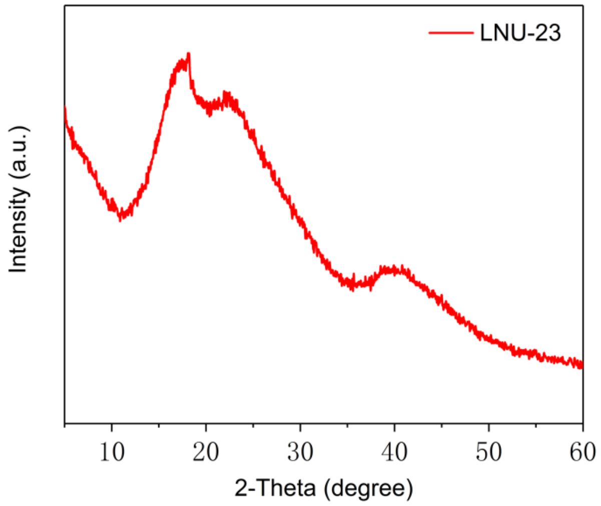

Figure S1. Powder X-ray diffraction pattern of LNU-23.

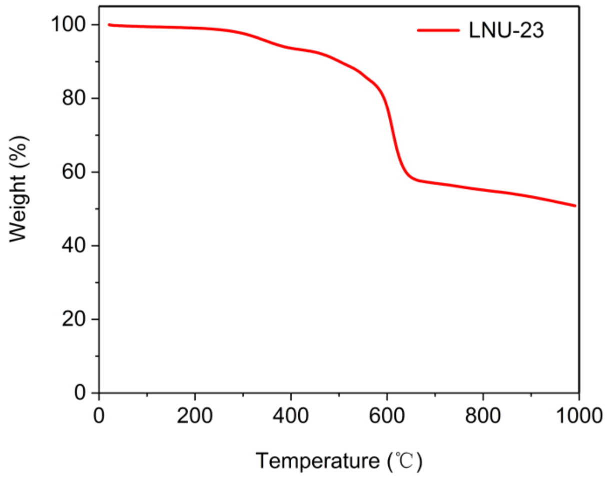

Figure S2. TGA plot of LNU-23 at N_2 condition.

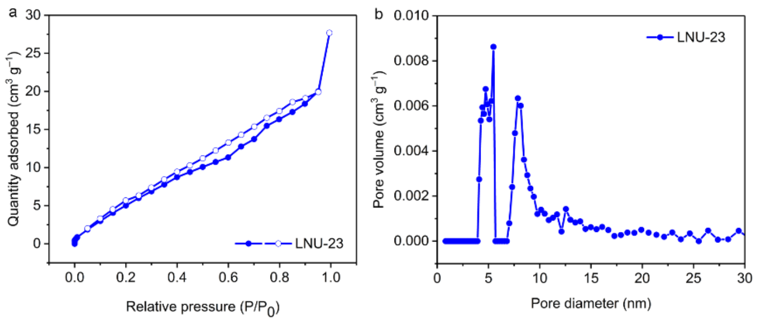

Figure S3. (a) N_2 adsorption-desorption isotherm of LNU-23; (b) Pore size distribution of LNU-23.

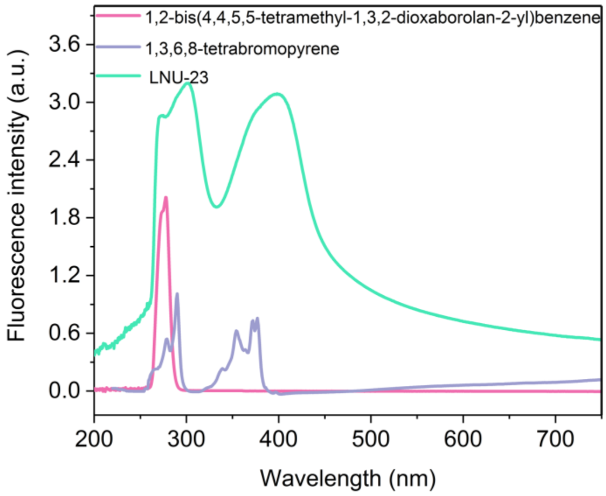

Figure S4. UV-Vis absorption spectra of 1,3,6,8-tetrabromopyrene, and 1,2-bis(4,4,5,5-tetramethyl-1,3,2-dioxaborolan-2-yl)benzene, and LNU-23.

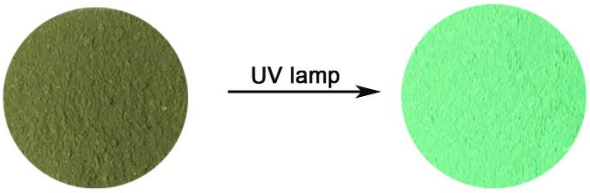

Figure S5. Luminescence photographs of LNU-23 powder under UV irradiation at 365 nm.

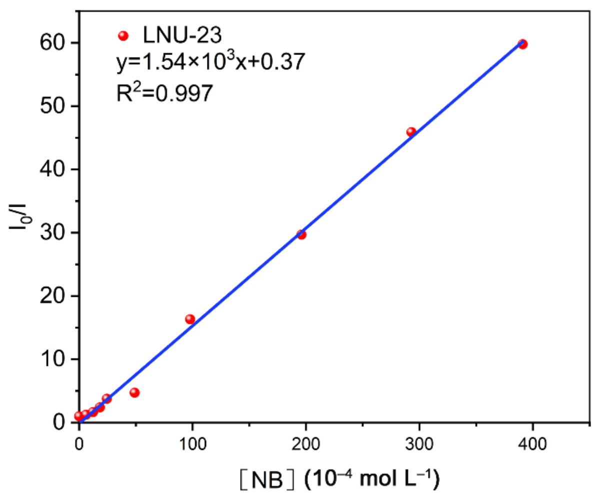

Figure S6. Stern-Volmer plot of LNU-23 treated with nitrobenzene at different concentrations.

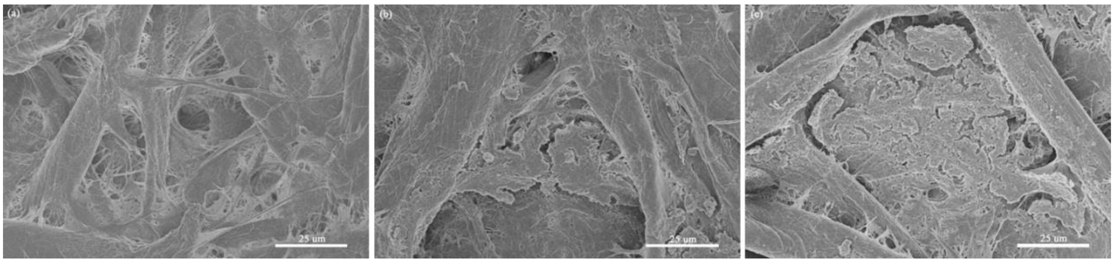

Figure S7. (a) SEM image of blank filter paper, (b) LNU-23 loaded paper, and (c) paper sensor soaked in absolute ethanol.

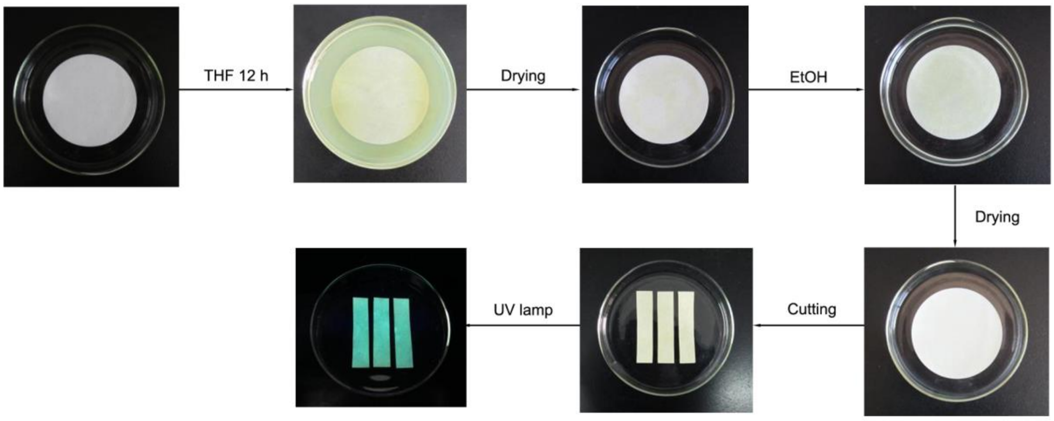

Figure S8. Preparation process of LNU-23 loaded paper sensor.

Table S1. Summary of luminescent probes for NB sensing.

Ln-MOFs	LOD	Ref
DF	8.12×10^{-4} M	[1]
BDS	4.06×10^{-4} M	
$NaCu_3(mtz)_4$	2.89×10^{-4} M	[2]
Cd-MOF	4.2×10^{-5} M	[3]
Eu^{3+}@SOF-1	3.7×10^{-5} M	[4]
$\{[Ln(dpc)(2H_2O)]\cdot(Hbibp)_{0.5}\}_n$	2.89×10^{-5} M	[5]
$[Zn\ (L)0.5(bpb)0.5(H_2O)_2]\ n$	2.0×10^{-5} M	[6]
$[Eu_2(L)_2(DMA)_2]_n\cdot nH_2O$	1.2×10^{-5} M	[7]
$[Zn(PBA)(pic)(H_2O)]\cdot H_2O\cdot 0.5DMA$	1.84×10^{-5} M	[8]
LNU-23	1.47×10^{-5} M	This work
M-15	9.91×10^{-6} M	[9]
$\{[Eu(L_2)_2(H_2O)_5]\cdot 3H_2O\}_n$	7.88×10^{-6} M	[10]
In/Tb-CBDA	8.88×10^{-6} M	[11]

References

1. Xie, H.L.; Wang, H.; Xu, Z.; Qiao, R.J.; Wang, X.F.; Wang, X.M.; Wu, L.F.; Lu, H.F.; Feng, S.Y. A silicon-cored fluoranthene derivative as a fluorescent probe for detecting nitroaromatic compounds. *J. Mater. Chem. C*, **2014**, *2*, 9425-9430.
2. Lu, Y.B.; Liao, Y.Q.; Dong, L.; Zhu, S.D.; Wen, H.R.; Huang, J.; Dai, X.X.; Lian, P.; Jiang, X.M.; Li, R.; Xie, Y.R. Ultra-stable metal-organic framework with concurrent high proton conductivity and fluorescence sensing for nitrobenzene. *Chem. Mater.* **2021**, *33*, 7858-7868.
3. Wang, X.L.; Liu, Yu.; Lin, H.Y.; Xu, N.; Liu, G.C.; Wang, X.; Chang, Z.H.; Li, J,R. A novel cadmium metal-organic framework-based multiresponsive fluorescent sensor demonstrating outstanding sensitivities and selectivities for detecting NB, Fe^{3+} ions and $Cr_2O_7^{2-}$ anions. *CrystEngComm*, **2020**, *22*, 6626-6631.
4. Jiang, D.Y.; Fang, H.F.; Li, G.; Zheng, G.L. A responsive supramolecular-organic framework: Functionalization with organic laser dye and lanthanide ions for sensing of nitrobenzene. *J Solid State. Chem.* **2020**, *284*, 121171-121177.
5. Yi, D.; Yang, H.; Liu, R.; Shao, C.; Yang, L. A multi-responsive chemosensor for highly sensitive and selective detection of Fe^{3+}, Cu^{2+}, $Cr_2O_7^{2-}$ and nitrobenzene based on a luminescent lanthanide metal-organic framework. *Dalton. Trans.* **2020**, *49*, 13003-13016.
6. Wang, L.B.; Wang, J.J.; Yue, E.L.; Li, J.F.; Tang, L.; Wang, X.; Hou, X.Y.; Zhang, Y.Q.; Ren, Y.X.; Chen, X.L. Luminescent Zn (II) coordination polymers for highly selective detection of triethylamine, nitrobenzene and tetracycline in water systems. *Dyes and Pigments*, **2022**, *197*, 109863-109872.
7. Wang, X.; Yan, P.; Li, Y.; An, G.; Yao, X.; Li, G. Highly efficient white-light emission and UV-visible/NIR luminescence sensing of lanthanide metal-organic frameworks. *Cryst. Growth Des.* **2017**, *17*, 2178-2185.
8. Xie, W.; Yuan, Y.; Zhou, T.Y.; Wang, J.J.; Nie, Z.Bin.; Xu, Y.H.; Su, Z.M. Stable zinc metal-organic framework as efficient bifunctional fluorescent probe for selective detection of nitrobenzene and Fe(III). *J. Solid State Chem.* **2022**, *310*, 123093-123099.
9. Quan, X.P.; Xu, X.; Yan, B. Facile fabrication of Tb^{3+}-functionalized COF mixed-matrix membrane as a highly sensitive platform for the sequential detection of oxolinic acid and nitrobenzene. *J. Haz. Mat.* **2022**, *427*, 127869-127878.
10. Sun, Z.; Sun, J.; Xi, L.; Xie, J.; Wang, X.; Ma, Y.; Li, L. Two novel lanthanide metal-organic frameworks: Selective luminescent sensing for nitrobenzene, Cu^{2+}, and MnO^{4-}. *Cryst. Growth Des*. **2020**, *20*, 5225-5234.
11. Zhang, Y.; Ying, Y.; Feng, M.; Wu, L.; Wang, D.; Li, C. Two isostructural Ln^{3+}-based heterometallic MOFs for the detection of nitro-aromatics and $Cr_2O_7^{2-}$. *New J. Chem.* **2020**, *44*, 12748-12754.
